# T-Cell Epitope Mapping of SARS-CoV-2 Reveals Coordinated IFN-γ Production and Clonal Expansion of T Cells Facilitates Recovery from COVID-19

**DOI:** 10.3390/v16071006

**Published:** 2024-06-22

**Authors:** Xing Fan, Jin-Wen Song, Wen-Jing Cao, Ming-Ju Zhou, Tao Yang, Jing Wang, Fan-Ping Meng, Ming Shi, Chao Zhang, Fu-Sheng Wang

**Affiliations:** 1 Senior Department of Infectious Diseases, The Fifth Medical Center of PLA General Hospital, National Clinical Research Center for Infectious Diseases, Beijing 100039, China; fanxing302@aliyun.com (X.F.); songjinwenchina@yeah.net (J.-W.S.); caowenjingjune@163.com (W.-J.C.); zhoumj89@163.com (M.-J.Z.); y_t_0321@163.com (T.Y.); wjing325@163.com (J.W.); drmengfanping@126.com (F.-P.M.); shiming302@sina.com (M.S.); 2 Medical School of Chinese PLA, Beijing 100853, China; 3 The First Affiliated Hospital of USTC, Division of Life Sciences and Medicine, University of Science and Technology of China, Hefei 230001, China

**Keywords:** SARS-CoV-2, COVID-19, T-cell epitopes, disease severity, IFN-γ, IL-17A, T-cell expansion

## Abstract

Background: T-cell responses can be protective or detrimental during severe acute respiratory syndrome coronavirus 2 (SARS-CoV-2) infection; however, the underlying mechanism is poorly understood. Methods: In this study, we screened 144 15-mer peptides spanning the SARS-CoV-2 spike, nucleocapsid (NP), M, ORF8, ORF10, and ORF3a proteins and 39 reported SARS-CoV-1 peptides in peripheral blood mononuclear cells (PBMCs) from nine laboratory-confirmed coronavirus disease 2019 (COVID-19) patients (five moderate and four severe cases) and nine healthy donors (HDs) collected before the COVID-19 pandemic. T-cell responses were monitored by IFN-γ and IL-17A production using ELISA, and the positive samples were sequenced for the T cell receptor (TCR) β chain. The positive T-cell responses to individual SARS-CoV-2 peptides were validated by flow cytometry. Results: COVID-19 patients with moderate disease produced more IFN-γ than HDs and patients with severe disease (moderate vs. HDs, *p* < 0.0001; moderate vs. severe, *p* < 0.0001) but less IL-17A than those with severe disease (*p* < 0.0001). A positive correlation was observed between IFN-γ production and T-cell clonal expansion in patients with moderate COVID-19 (r = 0.3370, *p* = 0.0214) but not in those with severe COVID-19 (r = −0.1700, *p* = 0.2480). Using flow cytometry, we identified that a conserved peptide of the M protein (Peptide-120, P120) was a dominant epitope recognized by CD8+ T cells in patients with moderate disease. Conclusion: Coordinated IFN-γ production and clonal expansion of SARS-CoV-2-specific T cells are associated with disease resolution in COVID-19. Our findings contribute to a better understanding of T-cell-mediated immunity in COVID-19 and may inform future strategies for managing and preventing severe outcomes of SARS-CoV-2 infection.

## 1. Introduction

Coronavirus disease 2019 (COVID-19), caused by severe acute respiratory syndrome coronavirus 2 (SARS-CoV-2), is associated with significant morbidity and mortality worldwide. With the global spread of SARS-CoV-2, the emergence of variants of concern (VOCs) poses significant challenges to epidemic control and development of vaccines and therapeutics [1]. For example, mutations in the spike protein in the Omicron strain, particularly within the Receptor Binding Domain (RBD), lead to a reduction in neutralizing antibody affinity by 36–40 times compared to the wild-type strain [2,3,4,5]. Moreover, the levels of SARS-CoV-2-specific B cells and neutralizing antibodies declined significantly over time in COVID-19 convalescents and vaccine recipients [6,7,8]. Thus, neutralizing antibodies offer limited protection against VOCs.

The antigen-specific T-cell response plays a critical role in clearing SARS-CoV-2 and controlling disease progression [9,10]. Early studies have shown that robust T-cell responses are associated with milder disease courses in COVID-19. Individuals with mild or asymptomatic COVID-19 have demonstrated significant T-cell responses despite having low antibody titers [11]. Moreover, the critical role of T-cell responses in controlling the virus has been shown in B-cell-depleted COVID-19 patients and SARS-CoV-2-infected non-human primate models [12,13,14]. However, dysregulation of the T-cell response has also been observed in severe COVID-19 cases. Evidence of increased total activated CD8+ T cells has been observed in some individuals with severe COVID-19, possibly indicating bystander CD8+ T-cell activation [15]. An imbalance between circulating regulatory and cytotoxic SARS-CoV-2-specific CD4+ T cells is associated with greater COVID-19 disease [16]. Therefore, a comprehensive investigation of the T-cell immune response in individuals with natural SARS-CoV-2 infection is crucial for understanding viral control and COVID-19 disease progression.

Over several hundred T-cell epitopes of SARS-CoV-2 have been described, and SARS-CoV-2 infection induces vigorous CD4+ and CD8+ T-cell responses, which exhibit remarkable breadth in most subjects [17]. Some mutated T-cell epitopes in SARS-CoV-2 VOCs were not recognized by CD8+ T cells in infected or vaccinated individuals, and some individuals even lost the CD8+ T-cell response to the Omicron strain [18,19]. Tarke et al. showed that more than 80% of T-cell reactivity was preserved at the population level and that, in most cases, no decrease was apparent [20]. These results indicate that the remarkable breadth of human T-cell responses makes it exceedingly difficult for variants to escape T-cell recognition at the population level [21]. However, the phenotypic diversity of epitope-specific T cells and their association with disease outcomes are poorly understood.

Thus, to study the T-cell response to SARS-CoV-2 antigens in COVID-19 patients and its correlation with disease severity, we synthesized 144 SARS-CoV-2 peptides covering spike, NP, M, ORF8, and ORF10 proteins, as well as 39 reported SARS-CoV-1 peptides. Subsequently, we screened their reactivity with peripheral blood mononuclear cells (PBMCs) of COVID-19 patients with moderate or severe disease, as well as healthy donors (HDs), by measuring IFN-γ and IL-17A production. We also analyzed T-cell expansion through T-cell receptor (TCR) sequencing and flow cytometry. Our results will improve our understanding of T-cell responses to SARS-CoV-2 infection.

## 2. Methods

### 2.1. Study Subjects

Thirteen laboratory-confirmed COVID-19 patients were hospitalized at the Fifth Medical Center of the Chinese PLA General Hospital in Beijing between 23 January and 26 May 2020, and nine healthy donors (HDs) were enrolled before the COVID-19 pandemic. All the subjects had no vaccination and no previous infection history. These patients were classified into two clinical groups, namely moderate and severe groups, according to the World Health Organization guidelines. Patients in the moderate disease group exhibited obvious clinical symptoms and pneumonia and were admitted to general wards but did not require intensive care. Patients in the severe disease group required critical care and met at least one of these criteria: dyspnea and respiratory rate ≥ 30/min; blood oxygen saturation ≤ 93%; PaO_2_/FiO_2_ ratio < 300 mmHg; lung infiltrates on CT scan >50% within 24–48 h; or who exhibited respiratory failure, septic shock, and/or multiple organ dysfunction/failure. Discharge criteria were consistent with the guideline, including the resolution of respiratory symptoms, substantial improvement in chest CT images, afebrile for more than 3 days, and two consecutive negative RT-PCR results from respiratory tract swab samples collected at least 24 h apart.

### 2.2. Sample Collection

Blood samples were collected from COVID-19 patients and HDs and centrifuged at 400× *g* for 5 min at room temperature. Plasma samples were stored at −80 °C until use. After plasma collection, PBMCs were isolated via Ficoll density gradient centrifugation and stored at −80 °C and in liquid nitrogen subsequently.

### 2.3. Epitopes Screening

One hundred and forty-four peptides spanning the spike (*n* = 83) and NP protein (*n* = 28) without overlap and the M (*n* = 7), ORF8 (*n* = 19), and ORF10 (*n* = 5) proteins with an 8-amino acid overlap were synthesized. Two ORF3a peptides were also synthesized. A total of 39 reported SARS-CoV-1 peptides were synthesized, including 17 spike protein peptides, 14 NP protein peptides, 7 M protein peptides, and 1 ORF3a protein peptide. The peptide sequence information is available in Table A1. All peptides were resuspended in DMSO and stored at −80 °C.

PBMCs were thawed and rested overnight in AIM medium (Gibco, New York, NY, USA) supplemented with 10% heat-inactivated human AB serum (Thermo Fisher Scientific, Waltham, MA, USA). PBMCs (1.5 × 10^5^) were seeded in a 96-well U bottom plate in AIM medium supplemented with 100 U/mL penicillin (Gibco), 0.1 mg/mL streptomycin (Gibco), 1000 IU/mL IL-2 (PeproTech, Rocky Hill, NJ, USA), 5 ng/mL IL-15 (PeproTech), 0.5 ng/mL IL-21 (PeproTech), and 1 µg/mL of one peptide (Genescript, Nanjing, China) and cultured at 37 °C in 5% CO_2_. An equimolar amount of DMSO was used to stimulate the cells as a negative control, and 1 mg/mL phytohemagglutinin (PHA, Roche, Basel, Switzerland) was used as the positive control. Heat-inactivated human AB serum was added the following morning to a final concentration of 5%. Seven days after incubation, the supernatant was collected and stored at −80 °C for enzyme-linked immunosorbent assay (ELISA). IFN-γ and IL-17A in the supernatant were assessed via ELISA (DAKEWE, Shenzhen, China) following the manufacturer’s instructions. The remaining cells were lysed using TRIzol reagent and stored at −80 °C for TCR sequencing and human leukocyte antigen (HLA) genotyping.

### 2.4. SARS-CoV-2 Specific Short-Term Cell Line Expansion and IFN-γ Expression Assessment via Flow Cytometry

PBMCs (1 × 10^5^) were stimulated with individual SARS-CoV-2 peptide in AIM medium with 5% AB serum and incubated at 37 °C in 5% CO_2_. IL-2 (10 U/mL; PeproTech) was added 4, 7, and 11 d after initial antigenic stimulation (1 μg/mL). On day 13, the culture medium was replaced with AIM containing 5% AB serum without any cytokines. On day 14, the short-term cell line was restimulated using SARS-CoV-2 peptides (10 μg/mL) for 6 h and GolgiPlug was added 5 h before harvest. For surface marker staining, T cells were stained with CD3-BV510, CD4-PerCP, and CD8-FITC antibodies (Biolegend, San Diego, CA, USA) for 30 min at 4 °C. For intracellular marker staining, cells were permeabilized using a Cytofix/Cytoperm Kit (BD Bioscience, Franklin Lakes, NJ, USA) and then stained with IFN-γ-PE-Cy7 antibody (Biolegend). Cells were fixed in 0.5% formaldehyde and analyzed by flow cytometry using a BD Canto II instrument and the data were analyzed using FlowJo software V10 (Tree Star Inc., Ashland, OR, USA).

### 2.5. Next-Generation Sequencing (NGS) of the TCR β Gene

Total RNA was extracted from each sample and subjected to RT-PCR amplification of the TCRβ gene using a QIAGEN one-step RT-PCR kit (QIAGEN, Hilden, Germany), with Cβ anti-sense primers and 36 Vβ mix primers used for two rounds of PCR. The resulting size-corrected PCR products were purified using magnetic beads (BioMagBeads, Wuxi, China). A sequencing library was prepared and purified using a Thermo Fisher Ion Plus Fragment Library Kit (Thermo Fisher Scientific), Ion Xpress Barcode Adaptors 1-16 Kit (Thermo Fisher Scientific), and Agencourt AMPure XP (Beckman Coulter, Brea, CA, USA). NGS was performed on an Ion S5 system using an Ion 530 Chip kit (Thermo Fisher Scientific).

### 2.6. TCR β Gene Sequence Analyses

The quality of the sequences obtained was monitored by cross-analyzing for potential contamination. Sequences shorter than 150 bp were excluded from analysis. TCR Vβ, Dβ, and Jβ germline gene assignment was conducted using a locally operating IgBlast program at Chengdu ExAb Biotechnology, Ltd. Non-functional TCR β VDJ sequences were removed, and 30,000 randomly selected TCR genes were analyzed.

### 2.7. HLA Genotyping

Genomic DNA (200 ng) from each sample was sheared using a Biorupter (Diagenode, Liège, Belgium) to acquire 150~200 bp fragments. The ends of the DNA fragments were repaired, and an Illumina Adaptor was added (Fast Library Prep Kit; iGeneTech, Beijing, China). After constructing the sequencing library, the target region was captured using an AI-HLA-Cap Enrichment Kit (iGeneTech) and sequenced on an Illumina platform (Illumina, San Diego, CA, USA) with 150 base paired-end reads. The raw reads were filtered using FastQC to remove low-quality reads. Clean reads were then mapped to the reference sequences in the HLA dictionary and typed to generate HLA types for HLA-A, -B, -C, -DPB1, -DQB1, and -DRB1 using HLA-HD Software (Version 1.5.0, Tokyo, Japan).

### 2.8. Peptide Homology Analysis

The P120 peptide sequences of different coronaviruses were obtained from the NCBI or Uniprot database, as follows: SARS-CoV-2 original strain M protein (P0DTC5), SARS-CoV-2 betta strain M protein (B.1.351, YP_009724393.1), SARS-CoV-2 delta strain M protein (B.1.617.2, QUX81285.1), SARS-CoV-2 Omicron Strain (B.1.1.529, UFO69282), SARS-CoV-1 M protein (P59596), 229E M protein(P15422), HKU1 M protein (Q5MQC7), NL63 M protein (K4P0W4), and OC43 M protein (YP_009555244.1). The CLUSTALW online tool was used to analyze the homology of P120 peptide among different coronaviruses (https://www.genome.jp/tools-bin/clustalw, accessed on 15 March 2024).

### 2.9. Ethical Approval

This study was approved by the Ethics Committee of the Fifth Medical Center of the Chinese PLA General Hospital (S2020-044-02). Informed consent was obtained from all enrolled participants or their legal guardians.

### 2.10. Statistical Analysis

GraphPad Prism statistical software (version 8.0; GraphPad Software, USA) and SPSS version 25 (SPSS Inc., USA) were used for statistical analysis. Spearman’s rank correlation was performed using the ‘ggcor’ package of the R software. Categorical variables were compared using the χ^2^ test or Fisher’s exact test and are reported as counts and percentages. Continuous variables were compared using the Mann–Whitney U test and Kruskal–Wallis H test and are presented as the median and interquartile range (IQR). Statistical significance was defined as a two-tailed *p*-value < 0.05.

## 3. Results

### 3.1. T Cells of Patients with Moderate Disease Had a Robust Response towards SARS-CoV-2 Antigens

**Figure 1:**
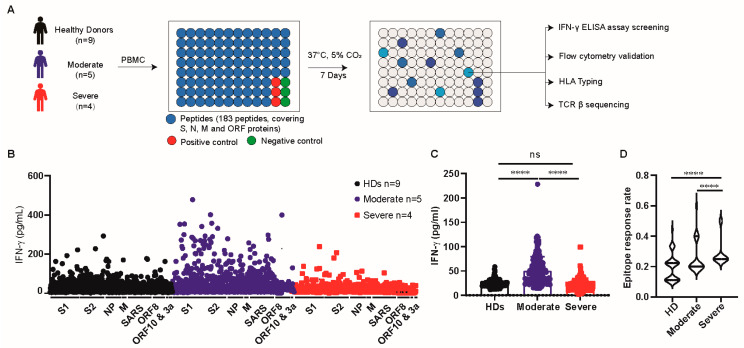
T cells of patients with moderate disease had a robust response towards SARS-CoV-2 antigens. (**A**) A schematic showing the overall study design. (**B**) IFN-γ in supernatant of cultured PBMCs from HD and COVID-19 patients with moderate or severe disease were assessed by ELISA, and antigen information was labeled along the X axis. (**C**) The mean value of IFN-γ concentration of cultured PBMCs from HD and COVID-19 patients with moderate or severe disease induced by each peptide assessed by ELISA. (**D**) Violin plot showing the response rate of each peptide in HD and COVID-19 patients with moderate or severe disease, two times higher IFN-γ concentration than the unstimulated sample was recognized as a positive response. S1: spike S1 domain; S2: spike S2 domain. Data are expressed as mean ± SD. and **** *p* < 0.0001 by two-tailed Mann–Whitney U-test. ns, non-significant.

**Figure A1:**
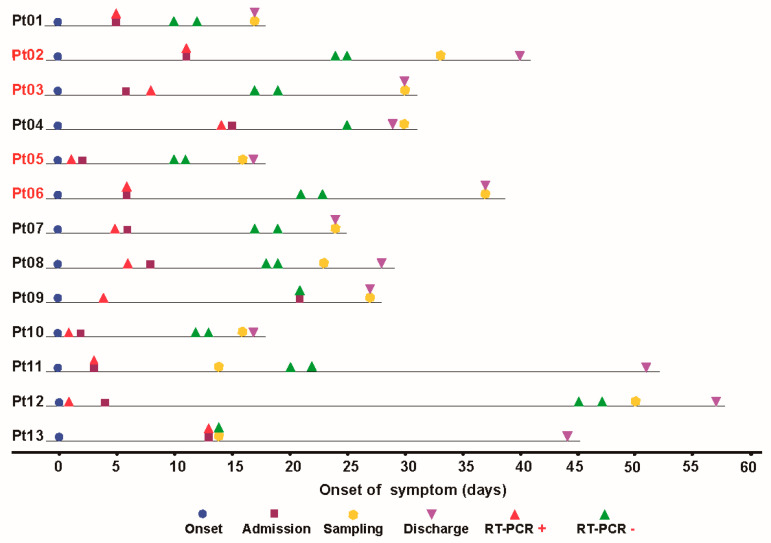
Timeline of the disease course for the enrolled 13 COVID-19 patients in our study. RT–qPCR indicates PCR test for SARS-CoV-2 nucleic acids. RT–qPCR positive indicates nasopharyngeal or sputum samples that were positive for SARS-CoV-2 nucleic acids.

To reveal the spectrum of T-cell responses to SARS-CoV-2 in naturally infected patients, we synthesized 144 peptides spanning the spike, NP, M, ORF8, ORF10, and ORF3a proteins. As the SARS-CoV-2 genome is highly homogenous with that of the SARS-CoV-1 virus, 39 reported SARS-CoV-1 peptides were synthesized (Table A1). Nine laboratory-confirmed and hospitalized COVID-19 patients (four severe and five moderate cases) were enrolled for epitope screening. Blood samples from nine HDs collected before the SARS-CoV-2 pandemic were used as controls. Demographic information and HLA alleles are listed in Table 1 and Table A2, and the timeline of the course of the disease is shown in Figure A1. PBMCs from HDs and COVID-19 patients were stimulated with each peptide and cultured for 7 days. IFN-γ in the supernatant was assessed using ELISA, and the experimental procedure is shown in Figure 1A. A positive response was defined as an IFN-γ concentration two-fold higher than that in the unstimulated sample. All samples that had a positive response were sequenced for the TCR β chain V-D-J gene usage and HLA allele. The IL-17A concentration in these positive samples was also analyzed using ELISA. The corresponding peptide responses were validated in short-term cells via flow cytometry.

**Figure A2:**
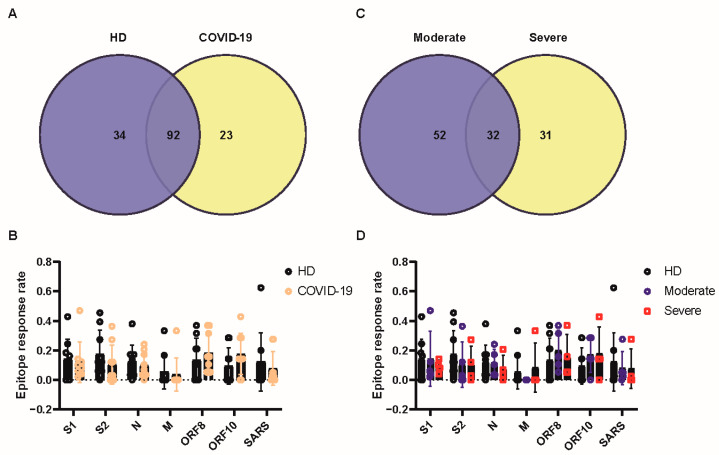
(**A**,**C**) Venn diagram illustrating the numbers of peptides recognized by COVID-19 patient and healthy donor cohorts (**A**), or by moderate and severe patients (**C**). (**B**,**D**) Response rate of SARS-CoV-2 peptides classified according to antigens in HDs and COVID-19 patients (**B**), or moderate and severe patients (**D**). SARS: SARS-CoV-1. Data are expressed as mean ± SD.

The IFN-γ secretion of PBMCs in patients with moderate COVID-19 was higher than that in patients with severe COVID-19 and HDs (moderate vs. HD, *p* < 0.0001; moderate vs. severe, *p* < 0.0001) (Figure 1B,C). Venn diagrams showed the number of responsive peptides detected in healthy donors (HDs), COVID-19 patients, as well as COVID-19 patients with moderate or severe disease. Of the 183 peptides, 92 exhibited reactivity in both HDs and COVID-19 patients, while 34 were recognized by HD and 23 were recognized by COVID-19 patients (Figure A2A). Notably, PBMCs from patients with moderate disease recognized more peptides than those from patients with severe disease (84 vs. 63) (Figure A2C). However, no obvious pattern of antigen specificity was observed between HDs and COVID-19 patients or between patients with moderate and severe disease (Figure A2B,D).

### 3.2. T-Cell Response of Patients with Severe Disease Produced Higher Levels of IL-17A

**Figure 2:**
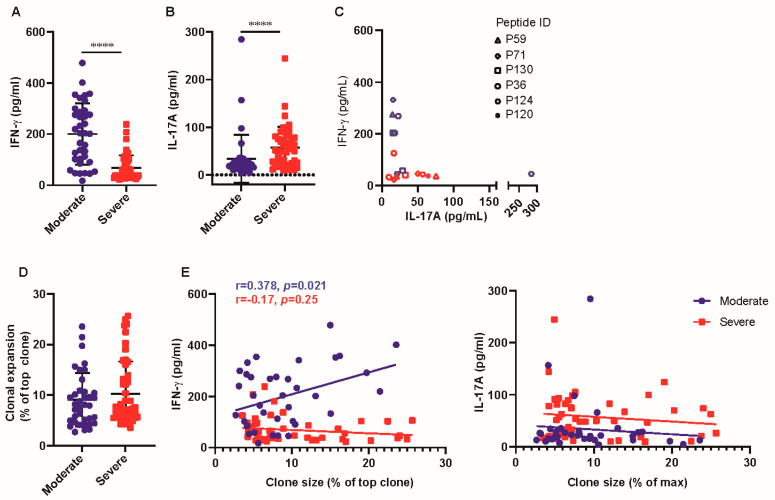
T-cell response of patients with severe disease produced higher levels of IL-17A. (**A**,**B**) IFN-γ (**A**) and IL-17A (**B**) of the positive-response sample was assessed by ELISA assay. (**C**) Spearman’s rank correlation between IFN-γ and IL-17A concentration in the supernatant of 6 representative samples was shown in dot plot. The dot shapes indicated representative peptides, the red indicated patients with severe disease, and the blue indicated patients with moderate disease. (**D**) The top TCR clone (immunodominant TCR β chain V-J gene pair) was analyzed between patients with moderate and severe disease. (**E**) Spearman’s rank correlation of the top TCR clone and IFN-γ or IL-17A was analyzed between patients with moderate and severe disease. Data are expressed as mean ± SD. **** *p* < 0.0001 by two-tailed Mann–Whitney U-test.

In the response-positive samples, the IFN-γ secreted by PBMCs of patients with moderate disease was higher compared to that of patients with severe disease (203.49 pg/mL [102.45, 286.08] vs. 50.22 pg/mL [33.25, 47.38]; *p* < 0.0001) (Figure 2A). As IL-17A is reported to be involved in COVID-19 disease progression [22], we analyzed the levels of IL-17A in the supernatant of positive samples. Interestingly, PBMCs from patients with severe disease expressed higher levels of IL-17A than those with moderate disease (50.03 pg/mL [22.91, 79.75] vs. 21.00 pg/mL [13.89, 29.93]; *p* = 0.0002) (Figure 2B). Then, we analyzed the bias of epitopes to induce IFN-γ or IL-17A production. The data in Figure 2C reveal a complex tendency in the secretion of IFN-γ or IL-17A induced by individual peptides, which is dependent on disease severity. IL-17A was more pronounced in patients with severe disease than in those with moderate disease.

To assess the antigen-specific T-cell expansion after antigen peptide stimulation, we sequenced the TCR β chain of the responsive sample and found that the proportion of the top immunodominant TCR β V-J gene pair (top TCR clone) was comparable between patients with moderate and severe disease (8.10% [4.97, 10.62] vs. 7.21% [5.62, 13.38], *p* = 0.47) (Figure 2D). The correlation between the proportion of top TCR clones and cytokine production was analyzed. The data in Figure 2E show a positive correlation between TCR expansion and IFN-γ production in patients with moderate disease (r = 0.38, *p* = 0.021) but not in those with severe disease (r = −0.17, *p* = 0.25) (Figure 2E, Left). No correlation between TCR expansion and IL-17A expression was observed in patients with moderate or severe disease (Figure 2E, Right). These data suggest that concerted IFN-γ production and TCR expansion in patients with moderate disease may favor disease resolution.

### 3.3. COVID-19 Patients Broadly Recognized Conserved P120 Peptide

**Figure 3:**
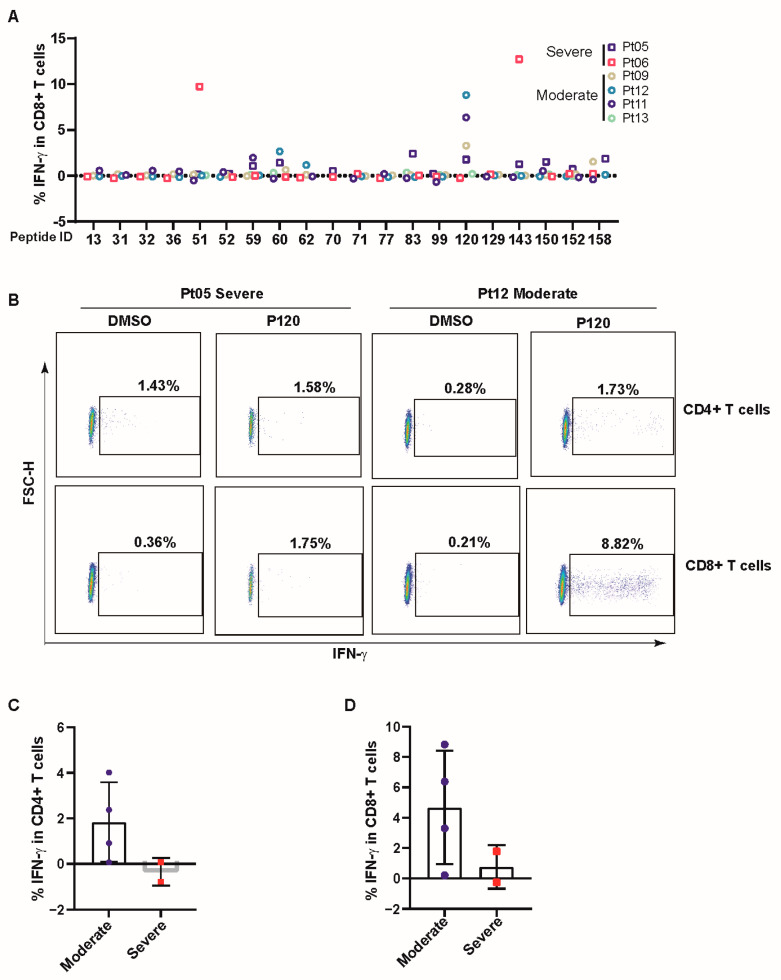
COVID-19 patients broadly recognized the conserved P120 peptide. (**A**) The short-term cell line was incubated with 20 peptides (10 ug/mL) for 5 h, and IFN-γ expression was measured by flow cytometry. (**B**) Representative flow cytometry results of IFN-γ expression in CD4+ or CD8+ T cells induced by P120 peptide in patients with moderate and severe disease. (**C**,**D**) The P120-specific CD4+ or CD8+ T-cell level was analyzed by flow cytometry. The value of antigen-specific T cells was calculated by subtracting the negative control. Data are expressed as mean ± SD.

We then validated the T-cell response towards SARS-CoV-2 peptides in short-term T cells of COVID-19 patients using flow cytometry. Twenty broadly recognized peptides identified (Table A4) in Figure 1D were validated in short-term T-cell lines from six COVID-19 patients, including four with moderate and two with severe disease. The demographic information and HLA alleles are listed in Table A2 and Table A3, and the timeline of the course of the disease is also shown in Figure A1. Compared to other peptides, the P120-peptide (P120) from the M protein (M_148–162_: HLRIAGHHLGRCDIK) elicited a broad and robust IFN-γ expression in CD8+ T cells in COVID-19 (Figure 3A). The P120 peptide was primarily recognized by CD8+ T cells of patients with moderate COVID-19 compared to those with severe COVID-19 (Figure 3B,C). It also induced a slight CD4+ T-cell response in patients with moderate COVID-19 (Figure 3D). Furthermore, the data in Figure 1B show that P120 induced higher IFN-γ secretion in the supernatant of PBMCs from patients with moderate disease. The median concentration of IFN-γ was 17.28 pg/mL in HDs, 64.78 pg/mL in patients with moderate disease, and 28.91 pg/mL in patients with severe disease. We used the Immune Epitope Database & Analysis Resource (IEDB) to predict potential epitopes within P120. Table A5 showed that P120 contains four epitopes (HLRIAGHHL, HLRIAGHHLGR, LRIAGHHLGR, and RIAGHHLGR) that could be presented by 15 HLA alleles. Combined analysis of IFN-γ production induced by P120 (assessed by ELISA or flow cytometry) and HLA alleles of P120-responsive patients revealed that HLA-A*30:01, HLA-B*13:02, HLA-B*46:01, HLA-B*08:01, HLA-C*01:02, HLA-C*03:03, and HLA-C*03:04 may present the P120 peptide. The epitope presented by these seven HLA alleles in P120 was the HLRIAGHHL peptide, which may contribute the CD8+ T-cell activation by P120 (Table A6). 

### 3.4. P120 Peptide Was Conserved among SARS-CoV-2 Variants and Induced Strong TCR Expansion

**Figure 4:**
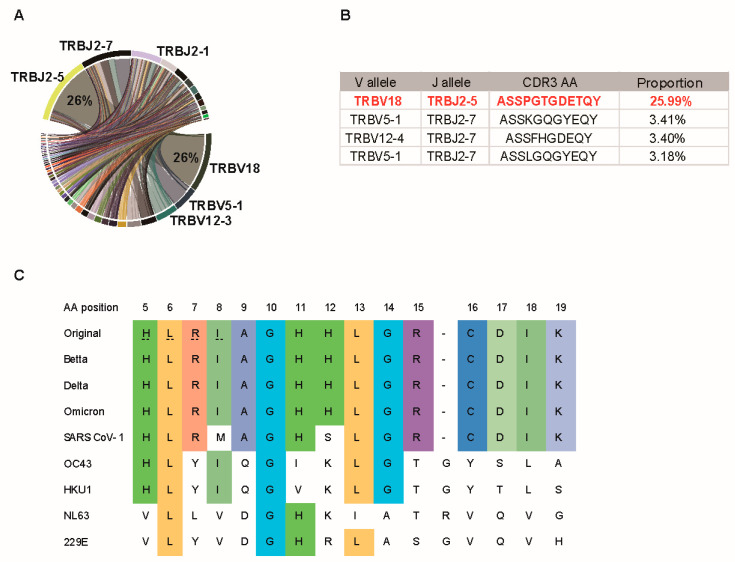
P120 peptide was conserved among SARS-CoV-2 variants and induces strong TCR expansion. (**A**) Circos plot depicting each β VJ gene usage of P120 responsive sample. (**B**) The proportion of the top 4 CDR3 sequences of the short-term cell of the P120 peptide. (**C**) Alignment of the amino acid sequences of original SARS-CoV-2 P120 peptides with Delta, Omicron variant, SARS-CoV-1, and endemic human coronaviruses.

**Figure A3:**
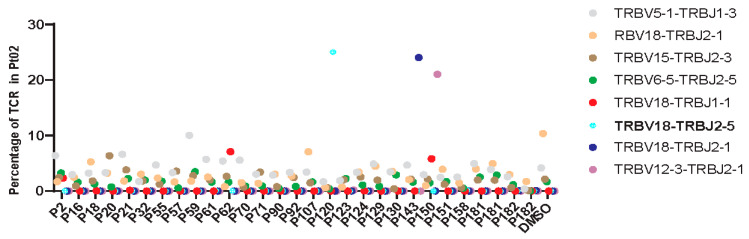
Eight dominant TCRs among all positive samples of Pt02 are presented. Different colors of the dot represent different TCRs as indicated on the right side of the panel.

To assess the VDJ gene usage of P120-responsive T cells, the TCR β chain of positive samples was sequenced. The result showed that in the P120-responsive samples of Pt02, the dominant D-J gene pair comprised TRBJ2-5 and TRBV18, constituting 25.99% of all D-J gene pairs (Figure 4A,B). We analyzed the level of the TRBV18-TRBJ2-5 TCR and seven other dominant TCRs in all the positive samples of Pt02, and the results showed that the levels of TRBV18-TRBJ2-5 TCR, TRBV18-TRBJ2-1 TCR, and TRBV12-3-TRBJ2-1 TCR were exclusively high in their corresponding samples than the other five dominant TCRs (Figure A3). This result indicated that TRBV18-TRBJ2-5 TCR, TRBV18-TRBJ2-1 TCR, and TRBV12-3-TRBJ2-1 TCR may be P120-, P143-, or P150-specific TCRs, respectively. Furthermore, the P120 peptide was not mutated among the reported SARS-CoV-2 variants (Figure 4C), rendering it a promising candidate for T-cell activation to confer protection against prevalent SARS-CoV-2 variants.

## 4. Discussion

In this study, we evaluated the reactivity of 183 SARS-CoV-2 and SARS-CoV-1 derived peptides towards PBMCs from nine COVID-19 patients and nine HDs. We found that T cells in patients with moderate disease had strong IFN-γ production with SARS-CoV-2 peptides, while patients with severe disease had higher IL-17A production. Although TCR amplification following antigen restimulation in vitro did not differ by COVID-19 disease severity, a positive correlation between TCR expansion and IFN-γ secretion was observed in patients with moderate but not severe disease. We identified one peptide (P120, M_148–162_) from the M protein broadly recognized by COVID-19 patients, especially those with moderate disease. The P120 peptide is conserved among SARS-CoV-2 variants, such as Delta and Omicron strains, and is presented by diverse HLA alleles. These results indicate that it is a promising target for promoting T-cell response and COVID-19 disease control.

Antigen-specific T cells are critical in the fight against viral infections. Associations between early T-cell responses and less severe COVID-19 outcomes have been observed [23,24]. We found that PBMCs in patients with severe disease produced less IFN-γ but higher levels of IL-17A compared to those of patients with moderate disease, despite similar levels of T-cell clone expansion between these two groups. Furthermore, a positive correlation was observed between the abundance of the top TCR β chain and IFN-γ production in patients with moderate disease, suggesting that coordinated IFN-γ production and clonal expansion of SARS-CoV-2-specific T cells are associated with disease control in COVID-19 patients. Conversely, such correlation was absent in severe cases, indicating that Th17 may be involved in the expanded T cells’ population, which may contribute to disease progression. COVID-19 patients with severe disease are characterized by inflammation and tissue damage in the respiratory tract, with monocyte and lymphocyte infiltration observed in the lungs [25,26,27,28]. Th17 cells characterized by high IL-17A and GM-CSF expression are enriched and clonally expanded in the lungs of COVID-19 patients with severe disease, potentially contributing to lung damage through interacting with CD8+ T cells and macrophages [22]. Additionally, IL-17A and Th17 cells are involved in the pathogenesis of MERS-CoV, SARS-CoV-1, and H1N1 infection [29,30,31].

Spike, NP, M, and NSP3 proteins are the major T-cell antigens of the SARS-CoV-2 virus, and differences exist in the phenotype and functional characteristics of T cells specific to different antigens [32]. Spike-specific T cells tend to differentiate into T follicular helper (TFH) cells, suggesting a crucial role in generating potent antibody responses, while M protein-specific and NP-specific CD4+ T cells are skewed toward a Th1 or Th1/Th17 profile [11]. Most studies have not distinguished the differences in T-cell responses induced by different SARS-CoV-2 antigens. We found that the IFN-γ and IL-17A production induced by the SARS-CoV-2 peptide was severity-dependent but not epitope-dependent. The bias of IL-17A production in patients with severe disease persisted even after several days of in vitro culture, and this phenomenon indicated that a stable program toward IL-17A production was established in patients with severe disease. This program may contribute to the elevated IL-17A in critical patients and be associated with fatal outcomes [22,33]. Furthermore, IL-17A has been implicated in promoting alveolar epithelial cell apoptosis and exacerbating the progression of pulmonary fibrosis, disrupting normal alveolar architecture and impairing alveolar–capillary gas exchange. Consequently, these processes adversely affect the normal oxygenation process, contributing to the respiratory symptoms [34].

After careful screening and validation, we identified a peptide (P120, M_148–162_: HLRIAGHHLGRCDIK) from the M protein, which was broadly recognized by COVID-19 patients with moderate disease and may contribute to disease control in SARS-CoV-2 infection. Similar to a prior study, a prevalent HLA-A*24:02-restricted epitope (M_198–206_) from the M protein was associated with convalescence in COVID-19 patients admitted to intensive care. While the reactivity of M_198–206_ was observed in all COVID-19 patients, CD8+ T cells specific to the M_198–206_ epitope exhibited an exhausted phenotype in severe cases compared to those in the moderate group [35]. The dysfunction of T cells in SARS-CoV-2 patients with severe disease has also been reported in the HLA-B*07:02-restricted NP_105–113_ epitope-responsive CD8+ T cells, which had a weaker response in patients who recovered from severe disease compared to those with mild disease [36]. The mechanism underlying the diminished responses of antigen-specific T cells in COVID-19 patients with severe disease remains unclear. It is hypothesized that the dysregulation of Tumor Necrosis Factor-α/Tumor Necrosis Factor Receptor 1 signaling, upregulation of inhibitory receptors, and prolonged antigen exposure may contribute to the dysfunction observed in antigen-specific T-cell responses in COVID-19 patients with severe disease [35,37,38].

Previous studies found that the P120 peptide could be presented by HLA-DRB1*04:04, HLA-DRB1*07:01, HLA-DRB1*11:01, or HLA-DRB1*15:01 alleles and promote CD4+ T-cell response [39,40]. However, in our study, four patients in the validation cohort with HLA-DRB1*15:01 or HLA-DRB1*11:01 alleles exhibited a very weak CD4+ T-cell expansion after being cultured with P120. Four epitopes within the P120 peptide (HLRIAGHHLGR, RIAGHHLGR, HLRIAGHHL, and LRIAGHHLGR) were predicted by the HLA binding algorithm and reported by other studies [41,42,43,44,45,46]. We found that the HLRIAGHHL peptide may be represented by HLA-A*30:01 and drive TRBV18-TRBJ2-5 TCR expansion in Pt02, consistent with another study [47].

The TCR β chain of a P120-responsive sample was sequenced, revealing that the predominant TCR V-J gene pair was TRBV18-TRBJ2-5, constituting 26% of the TCR β chains. TRBV18-TRBJ2-5 TCR was exclusively and prominently expanded in the P120-responsive sample compared with other positive samples in Pt02. We speculated that the HLRIAGHHL epitope in P120 might be presented by HLA-A*30:01 and drive the TRBV18-TRBJ2-5 TCR expansion in the in vitro culture assay. In a study on human immunodeficiency virus, researchers noted a significant TCR bias toward the HLA-B*0702-FPQGEAREL epitope, with TRBV18 and TRBJ2-5 being preferentially utilized. The TRBV18/CASSPRGREETQY/TRBJ2-5 clonotype retains functionality and persistence, even when antigen levels decay dramatically after antiretroviral therapy [48]. Therefore, the T cell of TRBV18-TRBJ2-5 expanded by the P120 peptide may preferentially persist under conditions of limited antigenic stimulation and provide long-term protection.

Our study had several limitations. While our peptide synthesis strategy avoided the bioinformatics bias of the epitope prediction algorithm, we only screened peptides derived from SARS-CoV-2 spike, NP, M, ORF8, and ORF10 proteins, with no overlap in spike and NP protein peptides. In addition, the epitopes of the ORF1a and ORF1b antigens were not screened. We only analyzed the correlation between TCR expansion and cytokine production. A comprehensive and detailed analysis of the relationship among HLA alleles, individual peptide responses, and TCR β chain sequences is needed to elucidate the comprehensive immune response of SARS-CoV-2. Moreover, our epitope screening included only nine COVID-19 patients and nine healthy donors (HDs), with significant variation in sampling time among COVID-19 patients. Additionally, glucocorticoid usage in COVID-19 patients with severe disease may weaken our conclusions. Our findings need to be validated in a larger cohort of COVID-19 patients.

## Figures and Tables

**Table 1:** Demographic characteristics of COVID-19 patients and HDs.

Patients	Gender	Age	Severity	Sampling (Day)	Sampling Status	Glucocorticoid before Sampling
Pt01	male	46	Moderate	17	discharge	No
Pt02	male	39	Severe	33	inpatient	Yes
Pt03	male	48	Severe	30	discharge	Yes
Pt04	male	48	Moderate	30	discharge	No
Pt05	female	85	Severe	16	discharge	No
Pt06	female	60	Severe	38	discharge	No
Pt07	male	37	Moderate	24	discharge	Yes
Pt08	female	44	Moderate	23	discharge	No
Pt10	female	48	Moderate	16	inpatient	No
HD-1	Male	29	NA	NA	HD	NA
HD-2	Male	30	NA	NA	HD	NA
HD-3	Female	27	NA	NA	HD	NA
HD-4	Female	27	NA	NA	HD	NA
HD-5	Female	28	NA	NA	HD	NA
HD-6	Male	28	NA	NA	HD	NA
HD-7	Male	26	NA	NA	HD	NA
HD-8	Female	26	NA	NA	HD	NA
HD-9	Female	25	NA	NA	HD	NA

Pt: patient.

## Data Availability

The original contributions presented in the study are included in the article, further inquiries can be directed to the corresponding author/s.

## Appendix A

**Table A1:** SARS-CoV-2 and SARS-CoV-1 peptides list.

Peptide ID	Name	Species	Antigen	Sequence	Length	Reference
P1	spike-1	SARS-CoV-2	Spike	MFVFLVLLPLVSSQC	15	
P2	spike-2	SARS-CoV-2	Spike	VNLTTRTQLPPAYTN	15	
P3	spike-3	SARS-CoV-2	Spike	SFTRGVYYPDKVFRS	15	
P4	spike-4	SARS-CoV-2	Spike	SVLHSTQDLFLPFFS	15	
P5	spike-5	SARS-CoV-2	Spike	NVTWFHAIHVSGTNG	15	
P6	spike-6	SARS-CoV-2	Spike	TKRFDNPVLPFNDGV	15	
P7	spike-7	SARS-CoV-2	Spike	YFASTEKSNIIRGWI	15	
P8	spike-8	SARS-CoV-2	Spike	FGTTLDSKTQSLLIV	15	
P9	spike-9	SARS-CoV-2	Spike	NNATNVVIKVCEFQF	15	
P10	spike-10	SARS-CoV-2	Spike	CNDPFLGVYYHKNNK	15	
P11	spike-11	SARS-CoV-2	Spike	SWMESEFRVYSSANN	15	
P12	spike-12	SARS-CoV-2	Spike	CTFEYVSQPFLMDLE	15	
P13	spike-13	SARS-CoV-2	Spike	GKQGNFKNLREFVFK	15	
P14	spike-14	SARS-CoV-2	Spike	NIDGYFKIYSKHTPI	15	
P15	spike-15	SARS-CoV-2	Spike	NLVRDLPQGFSALEP	15	
P16	spike-16	SARS-CoV-2	Spike	LVDLPIGINITRFQT	15	
P17	spike-17	SARS-CoV-2	Spike	LLALHRSYLTPGDSS	15	
P18	spike-18	SARS-CoV-2	Spike	SGWTAGAAAYYVGYL	15	
P19	spike-19	SARS-CoV-2	Spike	QPRTFLLKYNENGTI	15	
P20	spike-20	SARS-CoV-2	Spike	TDAVDCALDPLSETK	15	
P21	spike-21	SARS-CoV-2	Spike	CTLKSFTVEKGIYQT	15	
P22	spike-22	SARS-CoV-2	Spike	SNFRVQPTESIVRFP	15	
P23	spike-23	SARS-CoV-2	Spike	NITNLCPFGEVFNAT	15	
P24	spike-24	SARS-CoV-2	Spike	RFASVYAWNRKRISN	15	
P25	spike-25	SARS-CoV-2	Spike	CVADYSVLYNSASFS	15	
P26	spike-26	SARS-CoV-2	Spike	TFKCYGVSPTKLNDL	15	
P27	spike-27	SARS-CoV-2	Spike	CFTNVYADSFVIRGD	15	
P28	spike-28	SARS-CoV-2	Spike	EVRQIAPGQTGKIAD	15	
P29	spike-29	SARS-CoV-2	Spike	YNYKLPDDFTGCVIA	15	
P30	spike-30	SARS-CoV-2	Spike	WNSNNLDSKVGGNYN	15	
P31	spike-31	SARS-CoV-2	Spike	YLYRLFRKSNLKPFE	15	
P32	spike-32	SARS-CoV-2	Spike	RDISTEIYQAGSTPC	15	
P33	spike-33	SARS-CoV-2	Spike	NGVEGFNCYFPLQSY	15	
P34	spike-34	SARS-CoV-2	Spike	GFQPTNGVGYQPYRV	15	
P35	spike-35	SARS-CoV-2	Spike	VVLSFELLHAPATVC	15	
P36	spike-36	SARS-CoV-2	Spike	GPKKSTNLVKNKCVN	15	
P37	spike-37	SARS-CoV-2	Spike	FNFNGLTGTGVLTES	15	
P38	spike-38	SARS-CoV-2	Spike	NKKFLPFQQFGRDIA	15	
P39	spike-39	SARS-CoV-2	Spike	DTTDAVRDPQTLEIL	15	
P40	spike-40	SARS-CoV-2	Spike	DITPCSFGGVSVITP	15	
P41	spike-41	SARS-CoV-2	Spike	GTNTSNQVAVLYQDV	15	
P42	spike-42	SARS-CoV-2	Spike	NCTEVPVAIHADQLT	15	
P43	spike-43	SARS-CoV-2	Spike	PTWRVYSTGSNVFQT	15	
P44	spike-44	SARS-CoV-2	Spike	RAGCLIGAEHVNNSY	15	
P45	spike-45	SARS-CoV-2	Spike	ECDIPIGAGICASYQ	15	
P46	spike-46	SARS-CoV-2	Spike	TQTNSPRRARSVASQ	15	
P47	spike-47	SARS-CoV-2	Spike	SIIAYTMSLGAENSV	15	
P48	spike-48	SARS-CoV-2	Spike	AYSNNSIAIPTNFTI	15	
P49	spike-49	SARS-CoV-2	Spike	SVTTEILPVSMTKTS	15	
P50	spike-50	SARS-CoV-2	Spike	VDCTMYICGDSTECS	15	
P51	spike-51	SARS-CoV-2	Spike	NLLLQYGSFCTQLNR	15	
P52	spike-52	SARS-CoV-2	Spike	ALTGIAVEQDKNTQE	15	
P53	spike-53	SARS-CoV-2	Spike	VFAQVKQIYKTPPIK	15	
P54	spike-54	SARS-CoV-2	Spike	DFGGFNFSQILPDPS	15	
P55	spike-55	SARS-CoV-2	Spike	KPSKRSFIEDLLFNK	15	
P56	spike-56	SARS-CoV-2	Spike	VTLADAGFIKQYGDC	15	
P57	spike-57	SARS-CoV-2	Spike	LGDIAARDLICAQKF	15	
P58	spike-58	SARS-CoV-2	Spike	NGLTVLPPLLTDEMI	15	
P59	spike-59	SARS-CoV-2	Spike	AQYTSALLAGTITSG	15	
P60	spike-60	SARS-CoV-2	Spike	WTFGAGAALQIPFAM	15	
P61	spike-61	SARS-CoV-2	Spike	QMAYRFNGIGVTQNV	15	
P62	spike-62	SARS-CoV-2	Spike	LYENQKLIANQFNSA	15	
P63	spike-63	SARS-CoV-2	Spike	IGKIQDSLSSTASAL	15	
P64	spike-64	SARS-CoV-2	Spike	GKLQDVVNQNAQALN	15	
P65	spike-65	SARS-CoV-2	Spike	TLVKQLSSNFGAISS	15	
P66	spike-66	SARS-CoV-2	Spike	VLNDILSRLDKVEAE	15	
P67	spike-67	SARS-CoV-2	Spike	VQIDRLITGRLQSLQ	15	
P68	spike-68	SARS-CoV-2	Spike	TYVTQQLIRAAEIRA	15	
P69	spike-69	SARS-CoV-2	Spike	SANLAATKMSECVLG	15	
P70	spike-70	SARS-CoV-2	Spike	QSKRVDFCGKGYHLM	15	
P71	spike-71	SARS-CoV-2	Spike	SFPQSAPHGVVFLHV	15	
P72	spike-72	SARS-CoV-2	Spike	TYVPAQEKNFTTAPA	15	
P73	spike-73	SARS-CoV-2	Spike	ICHDGKAHFPREGVF	15	
P74	spike-74	SARS-CoV-2	Spike	VSNGTHWFVTQRNFY	15	
P75	spike-75	SARS-CoV-2	Spike	EPQIITTDNTFVSGN	15	
P77	spike-77	SARS-CoV-2	Spike	LQPELDSFKEELDKY	15	
P78	spike-78	SARS-CoV-2	Spike	FKNHTSPDVDLGDIS	15	
P79	spike-79	SARS-CoV-2	Spike	GINASVVNIQKEIDR	15	
P80	spike-80	SARS-CoV-2	Spike	LNEVAKNLNESLIDL	15	
P81	spike-81	SARS-CoV-2	Spike	QELGKYEQYIKWPWY	15	
P83	spike-83	SARS-CoV-2	Spike	TIMLCCMTSCCSCLK	15	
P84	spike-84	SARS-CoV-2	Spike	GCCSCGSCCKFDEDD	15	
P85	spike-85	SARS-CoV-2	Spike	SEPVLKGVKLHYT	13	
P86	nucleocapcid-1	SARS-CoV-2	NP	MSDNGPQNQRNAPRI	15	
P87	nucleocapcid-2	SARS-CoV-2	NP	TFGGPSDSTGSNQNG	15	
P88	nucleocapcid-3	SARS-CoV-2	NP	ERSGARSKQRRPQGL	15	
P89	nucleocapcid-4	SARS-CoV-2	NP	PNNTASWFTALTQHG	15	
P90	nucleocapcid-5	SARS-CoV-2	NP	KEDLKFPRGQGVPIN	15	
P91	nucleocapcid-6	SARS-CoV-2	NP	TNSSPDDQIGYYRRA	15	
P92	nucleocapcid-7	SARS-CoV-2	NP	TRRIRGGDGKMKDLS	15	
P93	nucleocapcid-8	SARS-CoV-2	NP	PRWYFYYLGTGPEAG	15	
P94	nucleocapcid-9	SARS-CoV-2	NP	LPYGANKDGIIWVAT	15	
P95	nucleocapcid-10	SARS-CoV-2	NP	EGALNTPKDHIGTRN	15	
P96	nucleocapcid-11	SARS-CoV-2	NP	PANNAAIVLQLPQGT	15	
P97	nucleocapcid-12	SARS-CoV-2	NP	TLPKGFYAEGSRGGS	15	
P98	nucleocapcid-13	SARS-CoV-2	NP	QASSRSSSRSRNSSR	15	
P99	nucleocapcid-14	SARS-CoV-2	NP	NSTPGSSRGTSPARM	15	
P100	nucleocapcid-15	SARS-CoV-2	NP	AGNGGDAALALLLLD	15	
P101	nucleocapcid-16	SARS-CoV-2	NP	RLNQLESKMSGKGQQ	15	
P102	nucleocapcid-17	SARS-CoV-2	NP	QQGQTVTKKSAAEAS	15	
P103	nucleocapcid-18	SARS-CoV-2	NP	KKPRQKRTATKAYNV	15	
P104	nucleocapcid-19	SARS-CoV-2	NP	TQAFGRRGPEQTQGN	15	
P105	nucleocapcid-20	SARS-CoV-2	NP	FGDQELIRQGTDYKH	15	
P106	nucleocapcid-21	SARS-CoV-2	NP	WPQIAQFAPSASAFF	15	
P107	nucleocapcid-22	SARS-CoV-2	NP	GMSRIGMEVTPSGTW	15	
P108	nucleocapcid-23	SARS-CoV-2	NP	LTYTGAIKLDDKDPN	15	
P109	nucleocapcid-24	SARS-CoV-2	NP	FKDQVILLNKHIDAY	15	
P110	nucleocapcid-25	SARS-CoV-2	NP	KTFPPTEPKKDKKKK	15	
P111	nucleocapcid-26	SARS-CoV-2	NP	ADETQALPQRQKKQQ	15	
P112	nucleocapcid-27	SARS-CoV-2	NP	TVTLLPAADLDDFSK	15	
P113	nucleocapcid-28	SARS-CoV-2	NP	QLQQSMSSADSTQA	14	
P114	membrane-1	SARS-CoV-2	M	MADSNGTITVEELKK	15	
P115	membrane-2	SARS-CoV-2	M	ITVEELKKLLEQWNL	15	
P116	membrane-3	SARS-CoV-2	M	KKLLEQWNLVIGFLF	15	
P117	membrane-4	SARS-CoV-2	M	EQWNLVIGFLFLTWI	15	
P118	membrane-5	SARS-CoV-2	M	LLESELVIGAVILRG	15	
P119	membrane-6	SARS-CoV-2	M	IGAVILRGHLRIAGH	15	
P120	membrane-7	SARS-CoV-2	M	HLRIAGHHLGRCDIK	15	
P121	SARS_2016.05.006	SARS-CoV-1	NP	LLNKHIDAYKTFP	13	PMID:27287409
P123	SARS_10.1128_2	SARS-CoV-1	Spike	NYNYKYRYLRGKLRPF	16	PMID:18832706
P124	SARS_10.1128_3	SARS-CoV-1	Spike	AGCLIGAEHVDTSYECDI	18	PMID:18832706
P125	SARS_10.1128_4	SARS-CoV-1	NP	GETALALLLLDRLNQ	15	PMID:18832706
P126	SARS_10.1128_5	SARS-CoV-1	M	GHLRMAGHSLGRCDI	15	PMID:18832706
P127	SARS_10.1128_6	SARS-CoV-1	Spike	NFNGLTGTGVLTPSSKRF	18	PMID:18832706
P128	SARS_10.1128_7	SARS-CoV-1	Spike	DIPIGAGICASYHTVSLL	18	PMID:18832706
P129	SARS_10.1128_8	SARS-CoV-1	Spike	SWFITQRNFFSPQII	15	PMID:18832706
P130	SARS_1	SARS-CoV-1	Spike	FIAGLIAIV	9	PMID: 15972696
P131	SARS_2	SARS-CoV-1	Spike	LITGRLQSL	9	PMID: 15972696
P132	SARS_3	SARS-CoV-1	Spike	RLNEVAKNL	11	PMID: 15972696
P133	SARS_4	SARS-CoV-1	Spike	ILPDPLKPT	9	PMID: 15016646
P134	SARS_5	SARS-CoV-1	Spike	VVFLHVTYV	9	PMID: 15016646
P135	SARS_6	SARS-CoV-1	Spike	KLPDDFMGCV	10	PMID: 16887973
P136	SARS_7	SARS-CoV-1	Spike	VLNDILSRL	9	PMID: 15016646
P137	SARS_8	SARS-CoV-1	NP	LLLDRLNQL	9	PMID: 16887973
P138	SARS_9	SARS-CoV-1	NP	RLNQLESKV	9	PMID: 16887973
P139	SARS_10	SARS-CoV-1	NP	GMSRIGMEV	9	PMID: 16887973
P140	SARS_11	SARS-CoV-1	NP	WLTYHGAIKLDDKDPQF	17	PMID: 15528730
P141	SARS_12	SARS-CoV-1	NP	QFKDNVILLNKHIDAYK	17	PMID: 15528730
P142	SARS_13	SARS-CoV-1	NP	MASGGGETALALLLLDRL NQLESKV	25	PMID: 17183651
P143	SARS_14	SARS-CoV-1	NP	TWLTYHGAIKLDDKDPQF KDNVILL	25	PMID: 17183651
P144	SARS_15	SARS-CoV-1	NP	GETALALLLL	10	PMID: 21813600
P145	SARS_16	SARS-CoV-1	M	LVIGFLFLAWIMLLQFAYS NRNRF	24	PMID: 17183651
P147	SARS_18	SARS-CoV-1	M	ILLNVPLRGTIVTRPLME SELVIG	24	PMID: 17183651
P148	SARS_19	SARS-CoV-1	M	IGNYKLNTDHAGSNDN IALLV	21	PMID: 17183651
P149	SARS_20	SARS-CoV-1	M	GHLRMAGHPLGRCDI	15	PMID: 17183651
P150	SARS-21	SARS-CoV-1	ORF3a	PLQASLPFGWLVIGV	15	PMID:18832706
P151	SARS_01026-X	SARS-CoV-1	Spike	DVNCTDVSTAIHADQLTP AWR	21	PMID: 15629033
P152	SARS_01025-8_1	SARS-CoV-1	NP	KDKKKKTDEAQPLPQRQ KKQ	20	PMID: 15629033
P153	SARS_01025-8_2	SARS-CoV-1	NP	QRQKKQPTVTLLPAAD MDDFSRQ	23	PMID: 15629033
P154	SARS_HLA-DR0401_1	SARS-CoV-1	Spike	NAFNCTFEYISDAFSLDV	18	PMID: 19050106
P155	SARS_HLA-DR0401_2	SARS-CoV-1	Spike	YISDAFSLDVSEKSGNFK	18	PMID: 19050106
P156	SARS_HLA-DR0401_3	SARS-CoV-1	Spike	YLRHGKLRPFERDISNVP	18	PMID: 19050106
P157	SARS_HLA-DR0401_4	SARS-CoV-1	Spike	RPFERDISNVPFSPDGK	17	PMID: 19050106
P158	SARS_3726.2005_1	SARS-CoV-1	M	MADNGTITVEELKQLLE QWNLVIGFLFLAWI	31	PMID: 16081901
P159	SARS_3726.2005_2	SARS-CoV-1	M	LMESELVIGAVIIRGHLR MAGHPLGRCDIK	30	PMID: 16081901
P160	SARS_5314.2004_1	SARS-CoV-1	NP	NNNAATVLQLPQGTTLP KGFYAEGSR	26	PMID: 15528730
P161	SARS_5314.2004_2	SARS-CoV-1	NP	KTFPPTEPK	9	PMID: 15528730
P162	ORF8_1	SARS-CoV-2	ORF8	MKFLVFLGIITTVAA	15	
P163	ORF8_2	SARS-CoV-2	ORF8	GIITTVAAFHQECSL	15	
P164	ORF8_3	SARS-CoV-2	ORF8	AFHQECSLQSCTQHQ	15	
P165	ORF8_4	SARS-CoV-2	ORF8	LQSCTQHQPYVVDDP	15	
P166	ORF8_5	SARS-CoV-2	ORF8	QPYVVDDPCPIHFYS	15	
P167	ORF8_6	SARS-CoV-2	ORF8	PCPIHFYSKWYIRVG	15	
P168	ORF8_7	SARS-CoV-2	ORF8	SKWYIRVGARKSAPL	15	
P169	ORF8_8	SARS-CoV-2	ORF8	GARKSAPLIELCVDE	15	
P170	ORF8_9	SARS-CoV-2	ORF8	LIELCVDEAGSKSPI	15	
P171	ORF8_10	SARS-CoV-2	ORF8	EAGSKSPIQYIDIGN	15	
P172	ORF8_11	SARS-CoV-2	ORF8	IQYIDIGNYTVSCLP	15	
P173	ORF8_12	SARS-CoV-2	ORF8	NYTVSCLPFTINCQE	15	
P174	ORF8_13	SARS-CoV-2	ORF8	PFTINCQEPKLGSLV	15	
P175	ORF8_14	SARS-CoV-2	ORF8	EPKLGSLVVRCSFYE	15	
P176	ORF8_15	SARS-CoV-2	ORF8	VVRCSFYEDFLEYHD	15	
P177	ORF8_16	SARS-CoV-2	ORF8	EDFLEYHDVRVVLDF	15	
P178	ORF8_17	SARS-CoV-2	ORF8	DVRVVLDFI	9	
P179	ORF8_A	SARS-CoV-2	ORF8	GNYTVSCSPFTINCQ	15	
P180	ORF8_BC	SARS-CoV-2	ORF8	GNYTVSCLPFTINCQ	15	
P181	ORF10_1	SARS-CoV-2	ORF10	MGYINVFAFPFTIYS	15	
P182	ORF10_2	SARS-CoV-2	ORF10	AFPFTIYSLLLCRMN	15	
P183	ORF10_3	SARS-CoV-2	ORF10	SLLLCRMNSRNYIAQ	15	
P184	ORF10_4	SARS-CoV-2	ORF10	NSRNYIAQVDVVNFN	15	
P185	ORF10_5	SARS-CoV-2	ORF10	QVDVVNFNLT	10	
P186	ORF3a_AB	SARS-CoV-2	ORF3a	VQIHTIDGSSGVVNP	15	
P187	ORF3a_C	SARS-CoV-2	ORF3a	VQIHTIDVSSGVVNP	15	

## Appendix B

**Table A2:** COVID-19 patients’ HLA alleles.

Patients	HLA-A	HLA-B	HLA-C	HLA-DPB1	HLA-DRB1	HLA-DQB1
Pt01	A*11:01:01; A*01:01:01	B*40:01:02; B*37:01:01	C*07:02:01; C*06:02:01	DPB1*13:01:01; DPB1*04:02:01	DRB1*01:01:01G; DRB1*12:02:01	DQB1*03:01:01; DQB1*05:01:01
Pt02	A*01:01:01; A*30:01:01	B*15:17:01; B*13:02:01	C*07:01:02; C*06:02:01	DPB1*05:01:01G; DPB1*14:01:01	DRB1*13:02:01; DRB1*09:01:02	DQB1*03:03:02; DQB1*06:04:01G
Pt03	A*24:02:01; A*29:01:01	B*07:02:01; B*35:03:01	C*07:02:01; C*04:01:01	DPB1*10:01:01; DPB1*02:01:02	DRB1*15:01:01; DRB1*11:04:01G	DQB1*06:02:01; DQB1*03:01:01
Pt04	A*11:01:01; A*30:01:01	B*13:02:01; B*46:01:01G	C*06:02:01; C*01:02:01	DPB1*05:01:01; DPB1*17:01:01	DRB1*07:01:01; DRB1*09:01:02	DQB1*03:03:02; DQB1*02:02:01
Pt05	A*11:01:01; A*11:01:01	B*38:02:01G; B*15:01:01	C*07:02:01; C*04:01:01	DPB1*05:01:01; DPB1*02:01:02	DRB1*15:01:01; DRB1*12:02:01	DQB1*06:02:01; DQB1*03:01:01
Pt06	A*24:02:01; A*33:03:01	B*40:01:02; B*58:01:01	C*03:02:02; C*03:04:01	DPB1*04:01:01; DPB1*04:02:01	DRB1*15:01:01; DRB1*03:01:01	DQB1*05:02:01; DQB1*02:01:01G
Pt07	A*02:06:01; A*02:01:01	B*13:02:01; B*15:11:01G	C*03:03:01; C*03:04:01	DPB1*02:01:02; DPB1*17:01:01	DRB1*07:01:01; DRB1*09:01:02	DQB1*03:03:02; DQB1*02:02:01
Pt08	A*24:02:01; A*26:01:01	B*08:01:01; B*46:01:01G	C*07:02:01; C*01:02:01	DPB1*02:01:02; DPB1*02:01:02	DRB1*15:02:01; DRB1*03:01:01	DQB1*05:02:01; DQB1*02:01:01G
Pt09	A*02:07:01G; A*02:06:01	B*15:01:01; B*46:01:01G	C*01:02:01; C*08:01:01	DPB1*05:01:01; DPB1*04:02:01	DRB1*09:01:02; DRB1*09:01:02	DQB1*03:03:02; DQB1*03:03:02
Pt10	NA	NA	NA	NA	NA	NA
Pt11	A*01:01:01; A*26:01:01	B*40:01:02; B*40:01:02	C*07:02:01; C*03:04:01	DPB1*05:01:01; DPB1*05:01:01G	DRB1*11:01:01; DRB1*08:03:02	DQB1*06:01:01; DQB1*03:01:01
Pt12	A*02:06:01; A*24:02:01	B*40:02:01; B*51:01:01	C*14:02:01; C*03:03:01	DPB1*05:01:01; DPB1*02:01:02	DRB1*16:02:01; DRB1*11:01:01	DQB1*05:02:01; DQB1*03:01:01
Pt13	A*02:01:01; A*01:01:01	B*37:01:01; B*46:01:01G	C*01:03:01G; C*06:02:01	DPB1*05:01:01; DPB1*04:01:01	DRB1*09:01:02; DRB1*10:01:01	DQB1*03:03:02; DQB1*05:01:01

Pt: patient.

## Appendix C

**Table A3:** Demographic characteristics of COVID-19 patients for validation assay.

Patients	Gender	Age	Severity	Sampling (Day)	Sampling Status	Glucocorticoid before Sampling
Pt05	Female	85	Severe	16	discharge	No
Pt06	Female	60	Severe	38	discharge	No
Pt09	Male	37	Moderate	27	discharge	No
Pt11	Male	58	Moderate	14	inpatient	No
Pt12	Male	42	Moderate	50	inpatient	No
Pt13	Female	53	Moderate	14	inpatient	Yes

Pt: patient.

## Appendix D

**Table A4:** List of twenty broadly responsive peptides.

Peptide ID	Name	Species	Antigen	Sequence	Length
P13	spike-13	SARS-CoV-2	Spike	GKQGNFKNLREFVFK	15
P31	spike-31	SARS-CoV-2	Spike	YLYRLFRKSNLKPFE	15
P32	spike-32	SARS-CoV-2	Spike	RDISTEIYQAGSTPC	15
P36	spike-36	SARS-CoV-2	Spike	GPKKSTNLVKNKCVN	15
P51	spike-51	SARS-CoV-2	Spike	NLLLQYGSFCTQLNR	15
P52	spike-52	SARS-CoV-2	Spike	ALTGIAVEQDKNTQE	15
P59	spike-59	SARS-CoV-2	Spike	AQYTSALLAGTITSG	15
P60	spike-60	SARS-CoV-2	Spike	WTFGAGAALQIPFAM	15
P62	spike-62	SARS-CoV-2	Spike	LYENQKLIANQFNSA	15
P70	spike-70	SARS-CoV-2	Spike	QSKRVDFCGKGYHLM	15
P71	spike-71	SARS-CoV-2	Spike	SFPQSAPHGVVFLHV	15
P77	spike-77	SARS-CoV-2	Spike	LQPELDSFKEELDKY	15
P83	spike-83	SARS-CoV-2	Spike	TIMLCCMTSCCSCLK	15
P99	nucleocapcid-14	SARS-CoV-2	NP	NSTPGSSRGTSPARM	15
P120	membrane-7	SARS-CoV-2	M	HLRIAGHHLGRCDIK	15
P129	SARS_10.1128_8	SARS-CoV-1	Spike	SWFITQRNFFSPQII	15
P143	SARS_14	SARS-CoV-1	NP	TWLTYHGAIKLDDKD PQFKDNVILL	25
P150	SARS-21	SARS-CoV-1	ORF3a	PLQASLPFGWLVIGV	15
P152	SARS_01025-8_1	SARS-CoV-1	NP	KDKKKKTDEAQPLPQRQKKQ	20
P158	SARS_3726.2005_1	SARS-CoV-1	M	MADNGTITVEELKQ LLEQWNLVIGFLFLAWI	31

## Appendix E

**Table A5:** Predicted epitopes within P120 peptides.

Cat	Allele	Length	Peptide	Score	Rank
1	HLA-A*11:01	9	RIAGHHLGR	0.479526	0.33
2	HLA-A*30:01	9	RIAGHHLGR	0.276817	0.49
3	HLA-A*30:01	9	HLRIAGHHL	0.187564	0.84
4	HLA-A*33:03	9	RIAGHHLGR	0.462495	0.36
5	HLA-A*33:03	11	HLRIAGHHLGR	0.327884	0.62
6	HLA-A*33:03	10	LRIAGHHLGR	0.169875	1.4
7	HLA-B*07:02	9	HLRIAGHHL	0.369452	0.37
8	HLA-B*08:01	9	HLRIAGHHL	0.508222	0.15
9	HLA-B*13:02	9	HLRIAGHHL	0.167793	1.1
10	HLA-B*15:01	9	HLRIAGHHL	0.279509	0.68
11	HLA-B*46:01	9	HLRIAGHHL	0.123246	0.92
12	HLA-C*01:02	9	HLRIAGHHL	0.067542	0.92
13	HLA-C*03:02	9	HLRIAGHHL	0.058591	1.9
14	HLA-C*03:03	9	HLRIAGHHL	0.029497	1.4
15	HLA-C*03:04	9	HLRIAGHHL	0.029497	1.4
16	HLA-C*07:01	9	RIAGHHLGR	0.007038	1.8
17	HLA-C*07:02	9	RIAGHHLGR	0.011405	1.9
18	HLA-C*14:02	9	HLRIAGHHL	0.052757	1.3

## Appendix F

**Table A6:** HLA genotyping of P120-responsive patients.

Patients	HLA-A	HLA-B	HLA-C	DPB1	DRB1	DQB1	IFN-γ Level	Detection Assay
Pt11	A*26:01; A*01:01	B*40:01; B*40:01	**C*03:04**; C*07:02	DPB1*05:01; DPB1*05:01	DRB1*08:03; **DRB1*11:01**	DQB1*03:01; DQB1*06:01	6.37%	Flow cytometry
Pt12	A*24:02; A*02:06	B*51:01; B*40:02	**C*03:03**; C*14:02	DPB1*02:01; DPB1*05:01	**DRB1*11:01**; DRB1*16:02	DQB1*03:01; DQB1*05:02	8.82%	Flow cytometry
Pt02	A*01:01; **A*30:01**	B*15:17; **B*13:02**	C*07:01; C*06:02	DPB1*05:01; DPB1*14:01	DRB1*13:02; DRB1*09:01	DQB1*03:03; DQB1*06:04	32.13 pg/mL	ELISA
Pt08	A*26:01; A*24:02	**B*46:01**; **B*08:01**	**C*01:02**; C*07:02	DPB1*02:01; DPB1*02:01	DRB1*03:01; DRB1*15:02	DQB1*02:01; DQB1*05:02	64.78 pg/mL	ELISA

HLA alleles in bold and grey shadow represent predicted HLA alleles with P120 peptide binding activity.
